# Codon optimization regulates IgG3 and IgM expression and glycosylation in *N. benthamiana*

**DOI:** 10.3389/fbioe.2023.1320586

**Published:** 2023-12-06

**Authors:** Lin Sun, Somanath Kallolimath, Roman Palt, Florian Eminger, Richard Strasser, Herta Steinkellner

**Affiliations:** Department of Applied Genetics and Cell Biology, Institute of Plant Biotechnology and Cell Biology, University of Natural Resources and Life Sciences, Vienna, Austria

**Keywords:** codon usage, antibody, transcription and translation, glycosylation, *Nicotiana benthamiana*

## Abstract

Plants are being increasingly recognized for the production of complex human proteins, including monoclonal antibodies (mAbs). Various methods have been applied to boost recombinant expression, with DNA codon usage being an important approach. Here, we transiently expressed three complex human mAbs in Nicotiana benthamiana, namely one IgG3 and two IgM directed against SARS-CoV-2 as codon optimized(CO) and non-codon optimized (NCO) variants. qRT-PCR exhibited significantly increased mRNA levels of all CO variants compared to the non-codon optimized orthologues, in line with increased protein expression. Purified CO and NCO mAbs did not exhibit obvious biochemical differences, as determined by SDS-PAGE and antigen binding activities. By contrast, enhanced production selectively impacts on glycosite occupancy and N-glycan processing, with increased mannosidic structures. The results point to a careful monitoring of recombinant proteins upon enhancing expression. Especially if it comes to therapeutic application even subtle modifications might alter product efficacy or increase immunogenicity.

## 1 Introduction

In last decades recombinant proteins have become one of the most important biological drugs. Due to its diverse applications monoclonal antibodies (mAbs) are lead products (Kaplon et al., 2023) with demands of purified recombinant mAbs in the double-digit tonne-range (Ecker and Seymour, 2020; Kaplon et al., 2023). In this context academia and the biotech industry have focused on the development of high expression systems, with mammalian cells as major platform. Nevertheless, other systems like plants, have received a lot of attention, especially due to the advent of highly potent transient expression vectors (Eidenberger et al., 2022; Eidenberger et al., 2023). To boost heterologous expression numerous strategies were applied, with DNA codon usage being a critical factor. In many cases it is imperative to modify the codon to match the usage pattern of recipient species for proper protein expression, however increased expression does not seem to be a general finding, and numerous studies report little or no effect (Mauro, 2018).

Codon usage bias, the preference for certain synonymous codons, were previously thought to be silent. However, a growing body of evidence now shows that codon usage not only regulates gene expression but also protein conformation (Liu, 2020). It is hypothesized that the non-uniform decoding rate across mRNAs mediated by codon usage represents a “code” within genetic codons that promote co-translational protein folding. However, relatively little is known about other co/posttranslational modifications induced by increasing expression levels. A modification frequently observed at mAbs derived from improved mammalian cells is a dramatic alteration of N-glycosylation (Bydlinski et al., 2020; Kosuge et al., 2020).

In plants, systematic studies on the impact of codon optimized (CO) to product quality are rare. While some groups demonstrate increased recombinant protein expression others do not see such an outcome, or even report reverse effects reviewed recently by (Pegoraro et al., 2017; Webster et al., 2017; Liu et al., 2022). The potential impact of CO to biochemical or functional features of plant-produced recombinant proteins, including mAbs, is unknown.

This study aimed at the investigation of three complex human antibodies produced parallelly as codon- and non-codon optimized (NCO) variants in plants with SARS-CoV-2 IgG3 and IgM as models. The impact of CO to specific mRNA abundance and recombinant protein expression was monitored. Further, a possible impact of enhanced production on co/posttranslational features, like mAb N-glycosylation, was evaluated.

## 2 Materials and methods

### 2.1 Generation of IgG3 and IgM constructs

To generate IgG3 and IgM expression constructs, cDNA fragments corresponding to constant heavy (C_H_) for IgG3C_H1-3_ and IgMC_H1-4_ (Supplementary Figure S1) without variable heavy chain (V_H_) sequences were codon optimized for *N. benthamiana* (GeneArt Gene Synthesis, Thermo Fisher Scientific) (Supplementary Figure S2). DNA fragments of IgG3C_H_ and IgMC_H_ were cloned to MagnICON^®^ pICH26211 (Castilho and Steinkellner, 2016), a viral based expression vector carrying a barley α-amylase signal sequence for peptide secretion, to assemble the template constructs without V_H_ (IgG3C_H_ - V_H_ and IgMC_H_ - V_H_). Codon-optimized V_H_ sequences of *H4* (379 bp) and *P5C3* (369 bp) were grafted onto the template constructs using BsaI restriction sites (Marillonnet et al., 2005), resulting into full length CO heavy chain constructs of *H4IgG3 CO_HC* (1512 bp), *H4*IgM CO_HC (1740 bp), and P5C3IgM CO_HC (1731 bp). Non-codon optimized IgG3 constructs and previously codon plant adapted (also referred as to NCO) IgM constructs (Loos et al., 2014), and the corresponding codon optimized kappa light chains *H4_κLC and* P5C3_*κ*LC (Supplementary Figure S3) have been described previously (Kallolimath et al., 2021; Sun et al., 2021; Kallolimath et al., 2023). All constructs were transformed into *Agrobacteria tumefaciens* (strain GV3101 pMP90) and used for subsequent agroinfiltration experiments.

### 2.2 Expression and purification of IgG3 and IgM variants

*Nicotiana benthamiana* (XFKO), a β1, 2-xylosyltransferases and α1, 3-fucosyltransferases knockout line (Strasser et al., 2008; Kogelmann et al., 2023), were grown under the long-day (16 h light/8 h dark) conditions at 24 C, 60% humidity. To produce IgG3 and IgM antibodies, corresponding constructs carrying the heavy (*H4IgG3 CO/NCO_HC*, *H4IgM CO/NCO_HC and P5C3IgM CO/NCO_HC*) and light chain (*H4_κLC* and *P5C3_κLC*) were co-expressed. For agro-infiltration, recombinant bacterial strains carrying gene of interests were grown at 29 °C for overnight, centrifuged and resuspended in infiltration buffer (10 mM MES, pH 5.6; 10 mM MgSO_4_). Agroinfiltration mixes were set to final OD_600_ at 0.1 to deliver into 4–5 weeks old plant leaves. To improve the N-glycan occupancy either the oligosaccharyltransferase (OST) from *Leishmania major* a single OST domain 3 (LmSTT3D) or OST from *Leshmania donovani* (LdOST) were co-infiltrated with an OD_600_ of 0.05. Total soluble proteins (TSPs) were extracted from ground leaves harvested at 4 days post infiltration (dpi) with extraction buffer (0.5 M NaCl, 0.1 M Tris, 1 mM EDTA, 40 mM ascorbic acid; pH 7.4) in a ratio of 1:2 w/v (fresh leaf weight/buffer).

Recombinant IgG3 and IgM variants were purified by using protein G (Protein G SepharoseTM Fast Flow, GE Healthcare) and by POROS™ CaptureSelect™ IgM Affinity Matrix (Thermo Scientific™, Cat no: 1080025), respectively. The purifications were carried out as described in detail recently (Kallolimath et al., 2021; Kallolimath et al., 2023). Antibodies were eluted with 0.1 M Glycine/HCl (pH 2.5–3.0), immediately neutralized with 1 M Tris (pH 9.0) and dialyzed overnight against PBS (PH 7.0).

### 2.3 RNA isolation and qRT-PCR

To perform quantitative reverse transcriptase polymerase chain reaction (qRT-PCR), total RNA was extracted as described (Sun et al., 2020). Briefly, 30 mg either IgG3 or IgM infiltrated leaf materials were harvested at 4 dpi and Monarch Total RNA Miniprep Kit (NEB) were used to extract total RNA. cDNA was synthesized from 1 µg of RNA using Luna Universal Probe One-Step RT-qPCR Kits (NEB). qRT-PCR was carried out in a C1000 Touch Thermal Cycler equipped with the CFX96 Touch Real-Time PCR Detection System (Bio-Rad), using a GoTaq^®^ qPCR and RT-qPCR Systems (Promega). All steps were performed according to the manufacturer’s recommendation. Elongation factor 1α (Ef1α) was used as internal control. The primer sequences were listed in Supplementary Table S1. Means and standard errors were calculated and the statistical significance was evaluated using the GraphPad Version 9 (http://www.graphpad.com) software. The significance of the data was evaluated using the Student’s t*-*test.

### 2.4 SDS-PAGE and immunoblotting

5 µL of TSPs or ∼4 µg of purified protein was separated on a 12% SDS-PAGE followed by Coomassie Brilliant Blue (R 250) staining or used for Immunoblotting using anti-human IgG (Goat anti-hIgG-HRPO, Invitrogen 62–8,420) and (Goat anti-human IgM-Fcµ5-HRPO, Sigma Aldrich AP114P) at the dilution 1:5,000.

### 2.5 Glycopeptide analysis

The N-glycosylation profiles of the purified mAbs were determined by mass spectrometry (MS) as described previously (Kallolimath et al., 2021; Sun et al., 2021). Briefly, purified mAbs were digested in solution with trypsin for IgG3, trypsin and Glu C for IgM, further analyzed with an LC-ESI-MS system (Thermo Orbitrap Exploris 480). The possible glycopeptides were identified as sets of peaks consisting of the peptide moiety and the attached N-glycan varying in the number of HexNAc units, hexose, deoxyhexose, and pentose residues. Manual glycopeptide searches were performed using FreeStyle 1.8 (Thermo), deconvolution was done using the extract function. The peak heights roughly reflect the molar ratios of the glycoforms. Nomenclature according to Consortium for Functional Glycomics (http://www.functionalglycomics.org) was used.

### 2.6 Direct sandwich ELISA

Direct sandwich ELISAs using SARS-CoV-2 spike protein RBD (Wuhan strain) as antigen and HRP-conjugated mAb CR3022 as secondary antibody (1:15,000 blotting buffer) was performed. ELISA was carried out in detail as previously described (Kallolimath et al., 2023). Purified CO and NCO IgG3 and IgM mAbs were coated at the concentration 2.0 μg/mL (H4) and 0.5 μg/mL (P5C3) with 50 µL/well to 96 well microplates (Thermo fisher maxisorp, catlog No: M9410-1CS). Absorbance was measured at 450 nm (reference 620 nm) using a Tecan Spark^®^ spectrophotometer. All samples were analyzed at least in two technical replicates. EC_50_ values were calculated by non-linear regression of the blank-corrected data points based on a four-parametric log model with GraphPad Prism (version 9).

## 3 Results

### 3.1 Codon optimization of IgG3 and IgM

Agroinfiltration was used extensively over the last decade for the transient expression of recombinant proteins in *N. benthamiana*. This applies also to mAbs, especially to IgG1, with large variation in expression levels (Shanmugaraj et al., 2020; Chen, 2022; Esqueda et al., 2023). Comparably moderate levels were obtained by transient expression of other Ab isotypes and subtypes, like IgM and IgG3 (Loos et al., 2014; Kallolimath et al., 2020; Kallolimath et al., 2021; Sun et al., 2021; Kallolimath et al., 2023). To overcome this shortcoming, custom gene synthesis services (Thermo Fisher Scientific) were applied using two SARS-CoV-2 neutralizing mAbs, H4 and P5C3 as models. Both originally developed as IgG1 (Wu et al., 2020; Fenwick et al., 2021) were switched to IgG3 and IgM variants (Kallolimath et al., 2021; Kallolimath et al., 2023). By delivering recombinant bacterial strains carrying CO/NCO_HC of IgG3 and IgM, together with corresponding κLCs to *N. benthamiana* (Kogelmann et al., 2023), six mAb variants were produced: H4IgG3 CO/NCO, H4 IgM CO/NCO, and P5C3IgM CO/NCO.

### 3.2 Codon optimization enhances mRNA and protein expression of IgG3 and IgM

**FIGURE 1:**
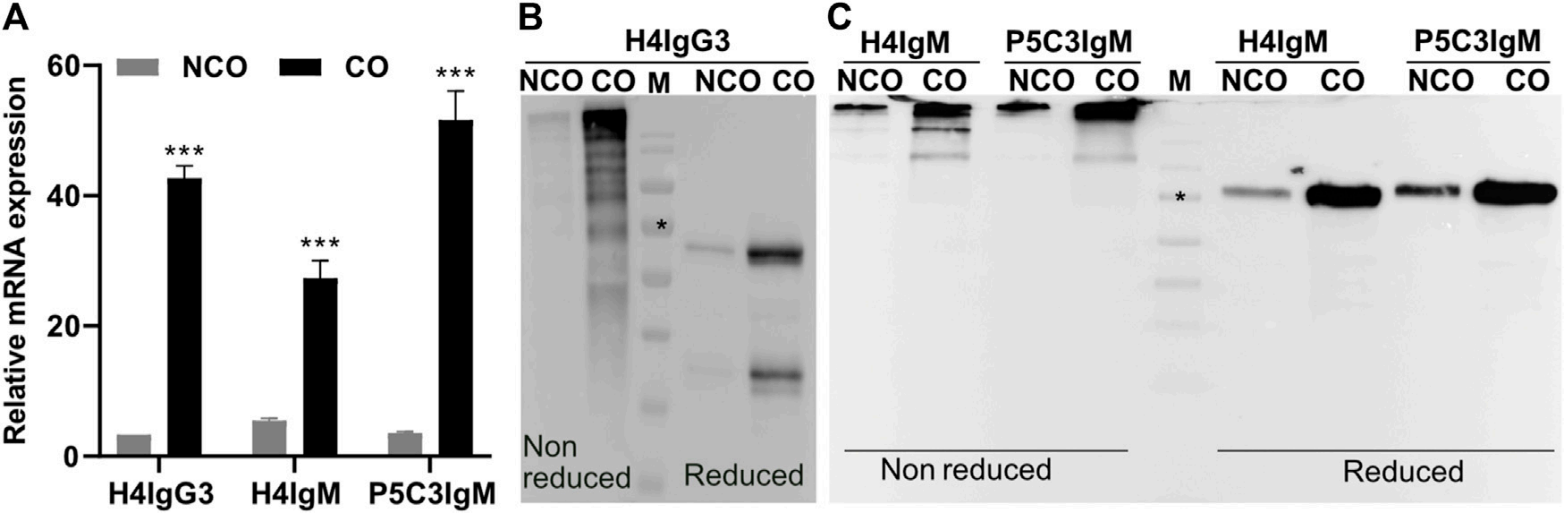
Codon optimization results in increased levels of both mRNA and protein of H4IgG3 and H4/P5C3IgM in *Nicotiana benthamiana*. **(A)**: Quantitative RT-PCR results showing the relative mRNA levels of IgG3 and IgM HC codon optimized (CO) and non-codon optimized (NCO). Western blot analysis of total soluble proteins (TSP) extracted from XFKO plants infiltrated with H4IgG3 **(B)** and H4/P5C3IgM **(C)** under reducing and non-reducing conditions (in each lane approx 50 µg TSP was loaded, Supplementary Figure S4, S5); M: marker: * = 70 kDa. Error bars shown in **(A)** are SDs of the means (n = 3). (***p < 0.001, Student’s t*-*test).

To monitor the effect of codon optimization on transcriptional level mRNA abundance of respective mAb HCs was assessed. Total RNA was extracted from leaves 4 dpi and subsequent qRT-PCR (Sun et al., 2020) revealed increased mRNA levels of CO-HCs compared to NCO counterparts for both, IgG3 and the two IgMs mAbs. The relative HC mRNA abundance increased about 15-fold (H4IgG3); 5- and 17-fold for H4IgM and P5C3IgM, respectively (Figure 1A).

Whether increased transcription activities of CO constructs impact on translation activities, in planta expression of recombinant mAbs was monitored. TSP extracted from agroinfiltrated leaves were subjected to SDS-PAGE followed by immunoblotting. Much stronger signals that refer to the corresponding CO-HCs were obtained, indicating an increased protein expression of these mAb compared to NCO variants (Figures 1B,C).

**FIGURE 2:**
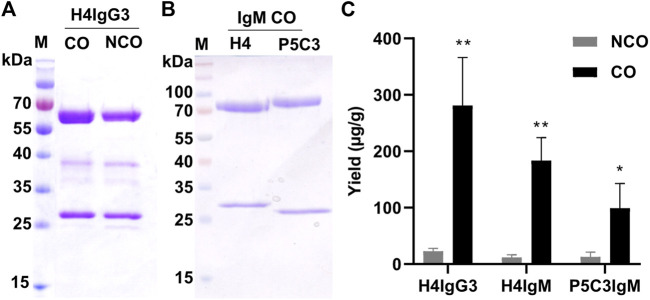
Codon optimization results in increased yields of purified IgG3 and IgM. SDS-PAGE (CBB stained) of purified H4IgG3 **(A)** and H4/P5C3IgM **(B)** under reducing conditions (4 µg protein was loaded at each lane. M: Marker. **(C)**: Yields (µg mAbs per g fresh leaf weight) of purified IgG3 and H4/P5C3IgM (Error bars shown are SDs of the means (n = 3). *p < 0.05, **p < 0.01, Student’s t*-*test).

Importantly, immunoblotting under non-reducing conditions exhibited efficient assembly of CO and NCO IgM mAbs, indicating that increased expression does not obviously affect assembly of mAbs (Figure 1C). For IgG3 variants, multiple bands were detected under non-reducing conditions and the precise assembly could not be determined (Figures 1B, Supplementary Figure S4). To monitor whether increased in planta expression has any effect on overall mAb yield, mAbs were subjected to affinity purification. Subsequent SDS-PAGE confirmed high purity of all mAbs and codon optimization did not cause unspecific or degraded bands that were not present in non-optimized Abs (Figures 2A,B). Importantly, purification yields of mAbs correlate to the immunoblotting signals (Figure 2C). The results indicate no adverse side effects using CO DNA, like imbalanced expression of HC or LC, that may lead to incomplete assembly. Overall, these results illustrate that codon optimization enhances both mRNA and protein levels of mAbs when transiently expressed in *N. benthamiana*.

### 3.3 Effect of codon optimization on antibody glycosylation and function

**FIGURE 3:**
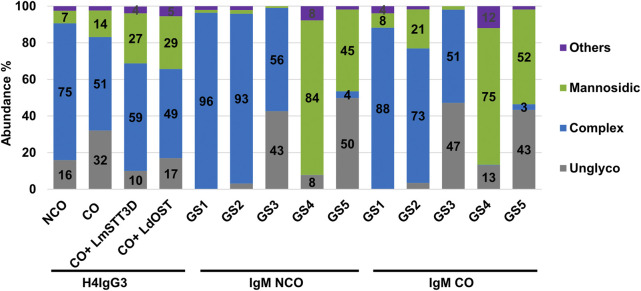
LC-ESI-MS–derived N-glycosylation profiles of purified IgG3 and IgM variants. Bars represent the relative abundance (%) of glycoforms detected at conserved Fc-GS of IgG3 and GS1-5 of IgM (for further details see Supplementary Table S2). Gray: unglycosylated; Blue: complex GlcNAc-terminating N-glycans; green: mannosidic N-glycans (Man4-Man9); purple: all other detected glycans combined.

Posttranslational modification (PTM), most importantly N-glycosylation, is a central quality parameter of recombinant mAbs. IgGs carry one conserved N-glycosite (GS) in the Fc domain, whereas human IgM glycosylation is more complex with five GSs in HC which are differentially occupied and carry individual glycan profiles (Supplementary Figure S1) (Loos et al., 2014; Kallolimath et al., 2023). In order to determine the N-glycosylation status of plant produced mAbs in detail, liquid chromatography-electrospray ionization-tandem mass spectrometry (LC-ESI-MS/MS) was performed. MS spectra of H4IgG3s displayed a single dominant glycoform at the Fc-GS, namely xylose and core fucose-free GlcNAc-terminated structures (predominantly GnGn), as typical for IgGs produced in these glycoengineered plants (Strasser et al., 2008; Kogelmann et al., 2023) (Figure 3 and Supplementary Table S2). In addition, mannosidic structures were detected, which were slightly higher in CO H4IgG3 (7% and 14%, respectively). Also, the CO variant showed increased unglycosylated Fc portions (∼32%) compared to the NCO IgG3 (∼16%). To overcome this unwanted side effect, single-subunit OSTs, which are able to increase IgG Fc glycosylation were co-expressed (Castilho et al., 2018; Beihammer et al., 2023). As expected, LmSTT3D and LdOST were able to significantly reduce the unglycosylated Fc-GS, down to levels observed with NCO IgG3 (Figure 3 and Supplementary Table S2).

Unlike IgG3, codon optimization of IgM had no obvious effects on the N-glycan occupancy. The glycosylation efficiency highly depends on individual GSs, a phenomenon often observed on proteins with multiple GSs (Arnold et al., 2005). While occupancy for GSs 1, 2 and 4 were almost 100%, GS3 and 5 were only partially glycosylated (∼50%). With regards to glycan profiles, GS1-3 carry mainly GnGn structures between 51%–96%, with GS4 and 5 exhibiting mainly mannosidic N-glycans between 45%–84% (Figure 3 and Supplementary Table S2). The results are in accordance with previously obtained outcomes (Loos et al., 2014; Kallolimath et al., 2023). Compared to NCO, CO IgMs exhibits increased mannosidic structures at GS1 and 2 (from 2 up to 21%).

**FIGURE 4:**
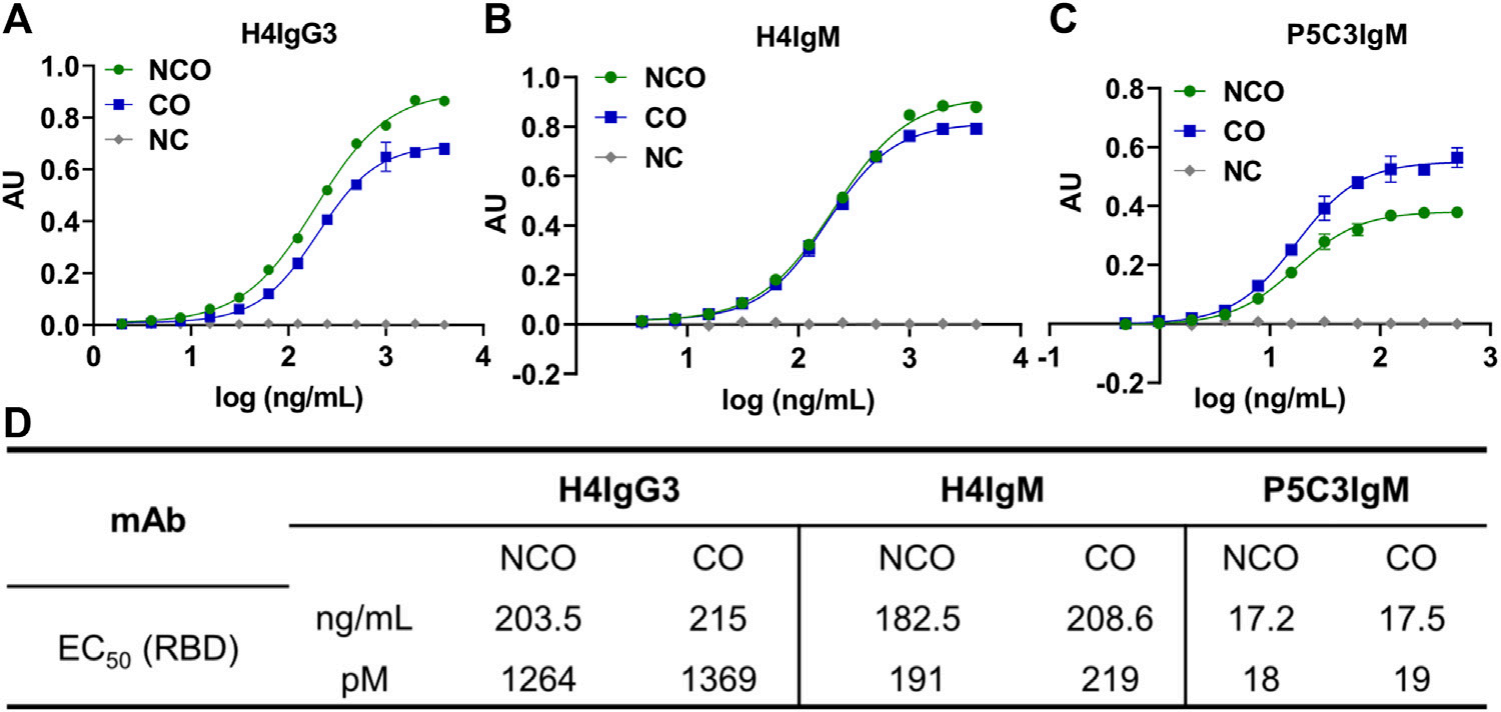
Codon optimization does not alter antigen-binding activity of IgG3 and IgMs. ELISA based antigen-binding activity of purified H4IgG3 **(A)** and H4/P5C3IgM **(B, C)** using recombinant RBD. X-axis: Log concentration (ng/mL); y-axis: absorbance (AU). **(D)**: EC_50_ values of tested antibodies in ng/mL and picomoles/L. NC: Negative control (irrelevant IgG1 mAb).

To evaluate a potential impact of codon optimization on the mAb’s function, an ELISA based antigen binding assay, using recombinant SARS-CoV-2 receptor binding domain was performed (Kallolimath et al., 2023). Irrespective of codon usage both antibody isotypes showed similar EC_50_ values (Figure 4) indicating no obvious differences in primary functional activities due to increased expression levels.

## 4 Discussion

Generally, most of the pharmaceutically relevant recombinant proteins expressed in plants are of human origin or from human pathogens. Therefore, the coding sequences of these proteins are likely to contain codons whose frequencies match human cellular activities rather than those in plants. While the impact of CO for heterologous expression has been reported controversially (Webster et al., 2017; Liu et al., 2022), we demonstrate consistent enhanced expression of all three CO mAbs compared to the NCO orthologues. In all cases improved protein expression is connected with HC mRNA abundance, although high levels of mRNA are not necessarily linked to high protein level (Vogel, 2013; Payne, 2015). A series of studies indicate that codon usage bias regulates gene expression at several stages, e.g. through mRNA stability, transcription and translation efficiency (recently reviewed by (Liu, 2020)). Our results indicate that RNA associated activities are the primary forces that drive enhanced expression in *N. benthamiana*.

Our outcomes demonstrate that increased protein expression does not obviously impact on the product integrity. Neither additional light/heavy chain associated degradation products nor apparent differences in mAb assembly was observed, in line with equivalent antigen binding of CO and NCO variants.

By contrast, we observed moderate differences in N-glycosylation an important PTM of Abs. IgG3 CO Fc-GS exhibited two alterations, increased mannosidic structures and decreased GS occupancy. Interestingly, while mannosidic structures rise on IgM GS1 and 2 no differences on GS occupancies were detected between CO and NCO versions. Currently we cannot clearly explain this observation. Obvious reasons are protein and/or GS specific differences. Another cause might be associated with expression levels and different demands for folding and assembly. Our results are in line with previous observations that report increased mannosidic structures and under-glycosylation in particularly high expressing IgG1 antibodies (Eidenberger et al., 2022). It seems that protein expression beyond a certain threshold impacts on N-glycosylation at various levels. This is in line with CHO produced mAbs, that show significantly altered glycosylation profiles due to increased expression (Stadlmann et al., 2008; Kosuge et al., 2020). Possible factors that contribute to this phenomenon are overloading the secretory pathway, insufficient ER associated quality control and/or shortage of components connected with the endogenous glycosylation machinery (Bydlinski et al., 2020). Here we confirm that under-glycosylation in plants might be addressed by the co-expression of OST components from other species (Castilho et al., 2018; Beihammer et al., 2023). Also, co-expression of chaperones can promote folding and reduce mannosidic N-glycans on recombinant glycoproteins (Margolin et al., 2020).

Collectively, in most cases codon optimization enhances mAb expression, and it is expected that associated methods based on algorithms will improve. However, with the wider experience also comes the realization that enhanced protein expression may result in PTM modifications that are currently unpredictable. This needs to be taken into account for product development. Especially if it comes to therapeutic application even subtle modifications as reported in this study might alter product function or increase immunogenicity.

## Data Availability

The datasets presented in this study can be found in online repositories. The names of the repository/repositories and accession number(s) can be found in the article/Supplementary Material.
