# Supplementary material for: Codon optimization regulates IgG3 and IgM expression and glycosylation in N. benthamiana
Source: Front Bioeng Biotechnol. 2023 Dec 6;11:1320586. doi: 10.3389/fbioe.2023.1320586 (PMC10731585; doi:10.3389/fbioe.2023.1320586)
Supplement: Supplementary file 1 [file DataSheet1.docx]

**Supplementary information for**

**Codon optimization regulates IgG3 and IgM expression and glycosylation in *N. benthamiana***

Lin Sun^1^, Somanath Kallolimath^1^, Roman Palt^1†^, Florian Emminger^1^, Richard Strassser^1^, Herta Steinkellner^1*^

^1^Institute of Plant Biotechnology and Cell Biology, Department of Applied Genetics and Cell Biology, University of Natural Resources and Life Sciences, Vienna, Austria

^†^ Current address: Virusure Forschung und Entwicklung, Donaucitystraße 1, 1220 Vienna

*Correspondence:

Herta Steinkellner

Email: [herta.steinkellner@boku.ac.at](mailto:herta.steinkellner@boku.ac.at)


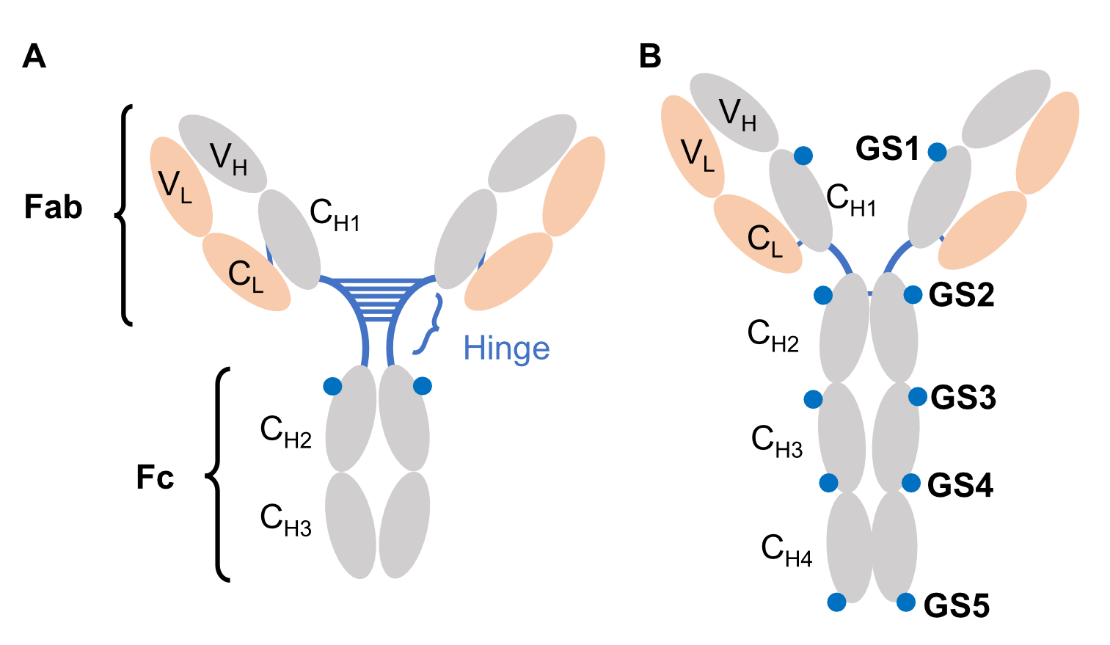


**FIGURE S1.**Diagrammatic structure of IgG3 and IgM including glycosites (GS).

The diagrammatic representation of IgG3 **(A)** and IgM **(B)** shows the variable and constant heavy (V_H_ and C_H_, respectively) and light chain (V_L_ and C_L_, respectively) domains. The antigen binding and crystallizable domains are represented as Fab and Fc respectively. Blue dots indicate conserved glycosites (GS), one at IgG3-Fc and 5 at IgM, GS1-5: ASN171 (NNS), 332 (NAS), 395 (NIS), 402 (NAT) and 563 (NVS).


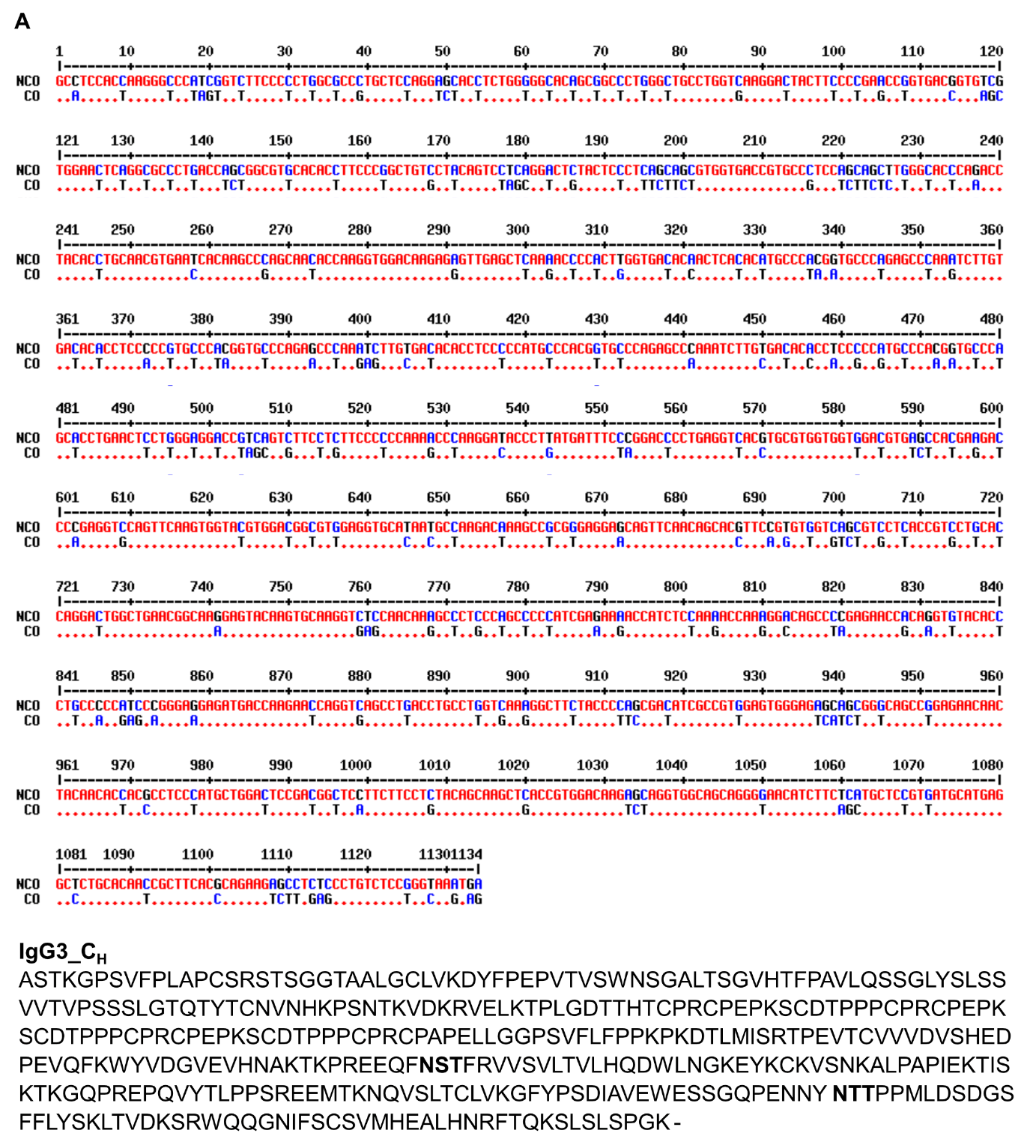


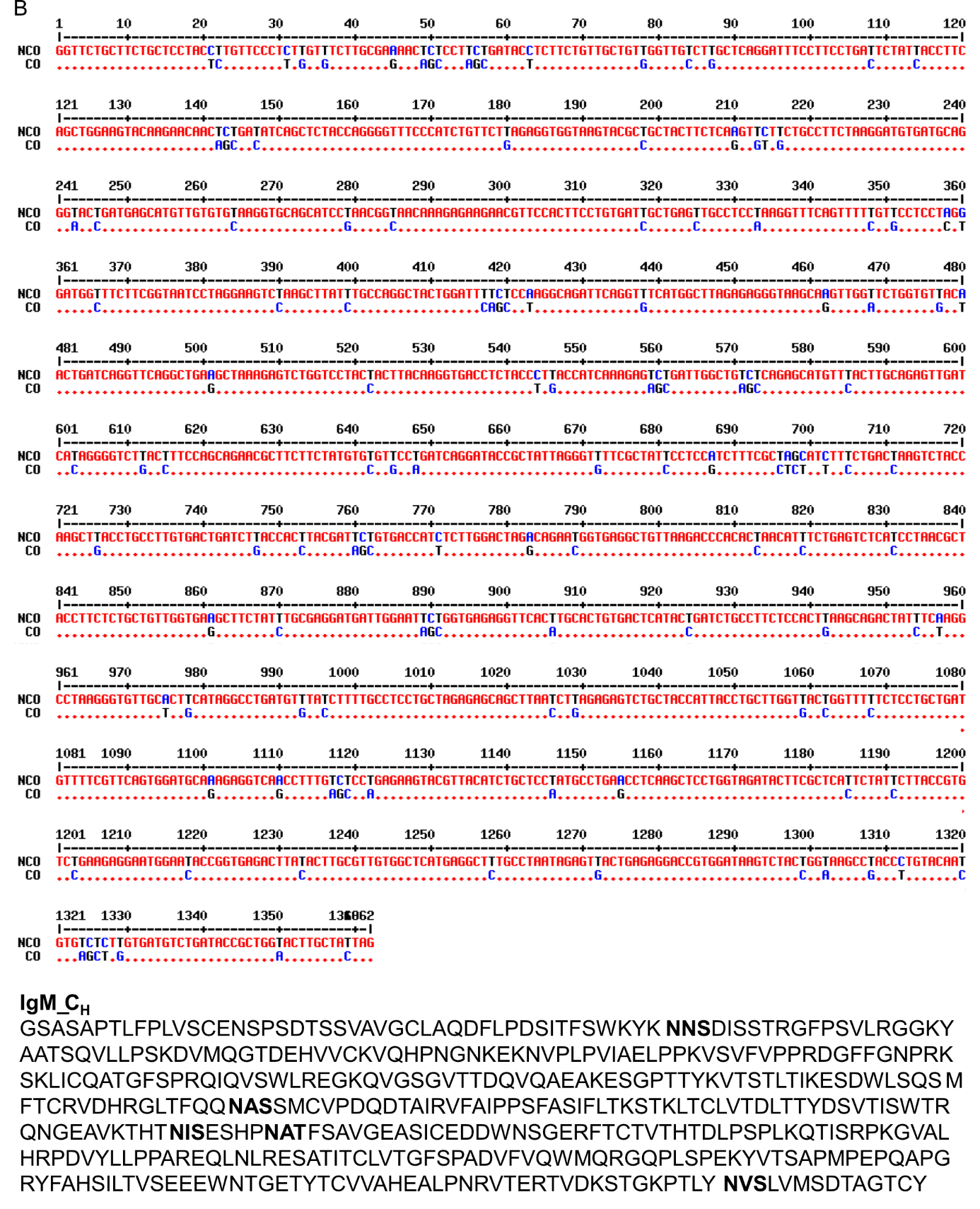


**FIGURE S2.** Comparison of IgG3 and IgM NCO and CO constant heavy chain (C_H_) DNA sequences and corresponding protein sequences.

**(A)**: Sequences of CO and NCO IgG3 _C_H_ (G3M5 allotype) DNA and protein. **(B)**: Sequences of CO and NCO IgM _C_H_ DNA and protein. Glycosites marked in bold, IgM GS1-5**:** NNS, NAS, NIS, NAT, NVS**.**

**
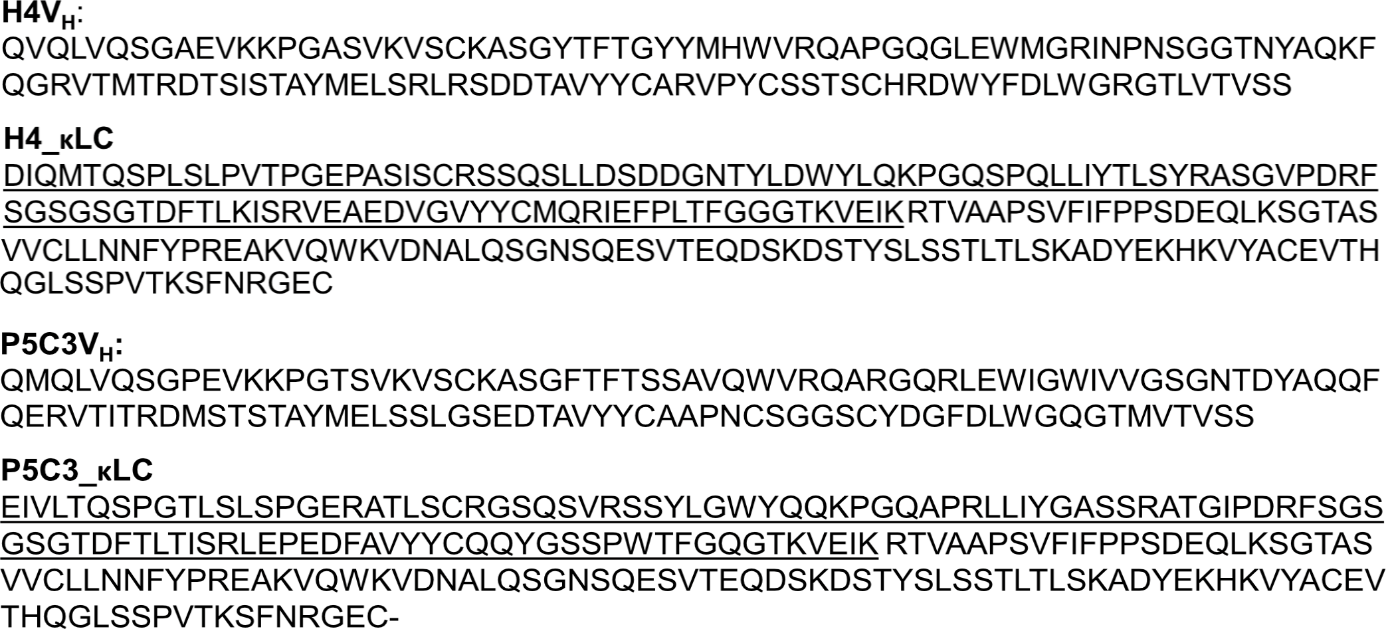
**

**FIGURE S3.** H4 and P5C3 variable heavy chain (H4V_H_ and P5C3V_H_) and kappa light chain (H4_κLC and P5C3_κLC) protein sequences.

Note: H4 and P5C3 variable light chains marked as underline.


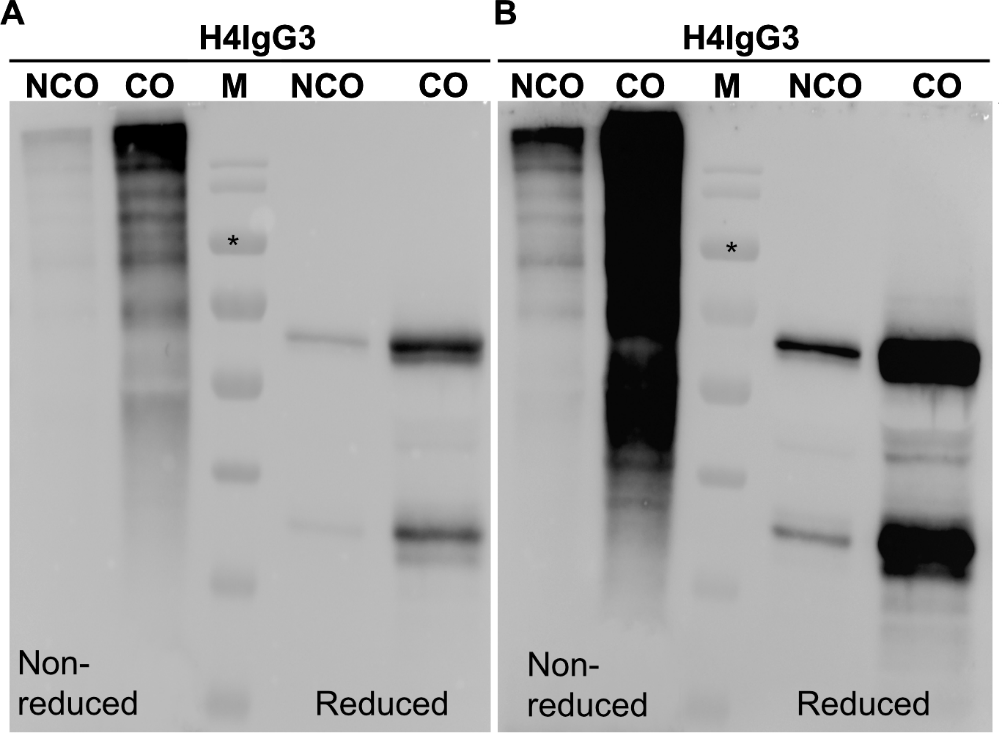


**FIGURE S4.** Codon optimization results in increased protein expression of IgG3 in *Nicotiana benthamiana.*

Western blot analysis of total soluble proteins (TSP) extracted from leaves infiltrated with H4IgG3 NCO and CO; **(A)**: 5s exposure time; **(B)**: 8 min exposure time. In each lane approx 50 µg protein was loaded; M: marker: ∗ = 70 kDa.


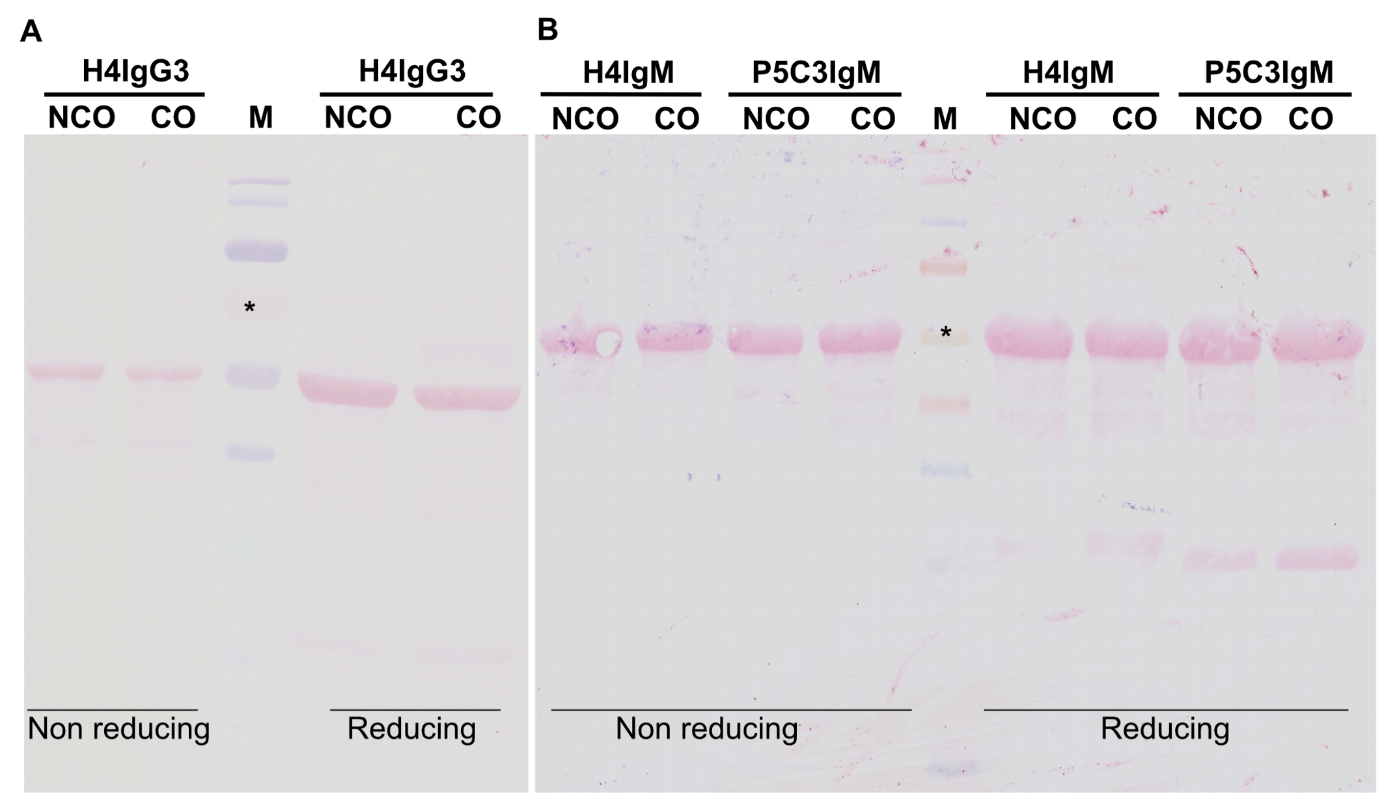


**FIGURE S5.** Ponceau S staining for loading control.

Corresponding Ponceau S-stained nitrocellulose membranes used for western blotting in Figure1 B and C demonstrating that equal protein amounts for the correlating samples were loaded. **A:** corresponds to Figure 1B; **B:** corresponds to Figure 1C; M: Marker, ∗ = 70 kDa.

**TABLE S1.** Primers used in this study.

| **qRT-PCR primers** | | **5´------3´** |
| --- | --- | --- |
| **H4 _forward** | **CTAACTACGCTCAGAAGTTCC** | |
| **H4 _reverse** | **ACCCCAAAGATCGAAGTACC** | |
| **P5C3_forward** | **ATACCGATTACGCCCAGCAG** | |
| **P5C3_reverse** | **AAACCATCGTAGCAAGAGCC** | |
| **Ef1α_forward** | **GCTGACTGTGCTGTCCTGATTATT** | |
| **Ef1α_reverse** | **TCACGGGTCTGTCCATCCTTA** | |

**TABLE S2.** The relative abundance of glycoforms (%) present at FC-GS of IgG3 and GS1-5 of IgM. Proglycan N-glycan nomenclature was used (https://homepage.boku.ac.at/jstadlmann/Proglycan_nomenclature_2023.pdf)

| **mAbs** | **GS** |  | **Unglyco** | **MGn** | **GnGn** | **Man4** | **Man5** | **Man6** | **Man7** | **Man8** | **Man9** | **MU** | **MM** | **Man4Gn** | **Man5Gn** | **Man9+1Hex** | **Sum** |
| --- | --- | --- | --- | --- | --- | --- | --- | --- | --- | --- | --- | --- | --- | --- | --- | --- | --- |
| **IgG3** |  | **NCO** | 15.9 | 9.8 | 64.9 | 0.4 | 0.8 | 0.4 | 1.0 | 2.1 | 2.2 | - | 0.6 | 1.3 | 0.4 | 0.2 | 100 |
|  |  | **CO** | 32.1 | 7.3 | 43.8 | 0.5 | 1.2 | 0.8 | 1.9 | 4.7 | 5.3 | - | 0.6 | 1.0 | 0.5 | 0.3 | 100 |
|  |  | **CO+ LmSTT3D** | 10.2 | 8.8 | 50.0 | 1.3 | 2.9 | 2.0 | 3.6 | 7.1 | 10.3 | - | 0.8 | 1.4 | 0.9 | 0.3 | 100 |
|  |  | **CO+ LdOST** | 17.0 | 5.7 | 43.0 | 2.7 | 4.3 | 3.0 | 4.1 | 6.7 | 8 | - | 2.6 | 1.5 | 1.2 | 0.3 | 100 |
| **IgM** | **GS1** | **NCO** | 0 | 81.5 | 14.9 | 0 | 0 | 0 | 0 | 1.6 | 0 | 0 | 1.3 | 0.7 | 0 | 0 | 100 |
|  |  | **CO** | 0 | 73.3 | 15 | 0 | 0 | 0 | 0 | 5.7 | 2.1 | 1.7 | 1.6 | 0.6 | 0 | 0 | 100 |
|  | **GS2** | **NCO** | 3.2 | 10.5 | 82.2 | 0.3 | 0.5 | 0 | 1.2 | 0 | 0 | 0.9 | 1.2 | 0 | 0 | 0 | 100 |
|  |  | **CO** | 3.2 | 10.5 | 82.2 | 0.3 | 0.5 | 0 | 1.2 | 0 | 0 | 0.9 | 1.2 | 0 | 0 | 0 | 100 |
|  | **GS3** | **NCO** | 42.6 | 1.4 | 54.9 | 0 | 0 | 0 | 0 | 0.6 | 0.3 | 0 | 0 | 0 | 0 | 0 | 100 |
|  |  | **CO** | 47.2 | 1.3 | 49.6 | 0 | 0 | 0 | 0 | 0.9 | 1 | 0 | 0 | 0 | 0 | 0 | 100 |
|  | **GS4** | **NCO** | 7.7 | 0.1 | 0 | 2.0 | 4.5 | 5.6 | 12.3 | 21.7 | 38.2 | 0 | 0.8 | 0 | 0.3 | 6.7 | 100 |
|  |  | **CO** | 13.2 | 0.1 | 0.1 | 3.4 | 7.7 | 10.8 | 19.7 | 32.3 | 0.7 | 0 | 1.2 | 0 | 0.5 | 10.3 | 100 |
|  | **GS4** | **NCO** | 49.8 | 0.7 | 3 | 0.5 | 1.3 | 3.9 | 20.8 | 13.1 | 5.1 | 0 | 0 | 0.5 | 1.1 | 0.3 | 100 |
|  |  | **CO** | 43.4 | 0.6 | 2.5 | 0.5 | 1.1 | 3.5 | 21.8 | 17.6 | 7.2 | 0 | 0 | 0.5 | 1 | 0.4 | 100 |
|  |  |  | **Unglyco** | **Complex** | | **Mannosidic** | | | | | | **Others** | | | | |  |
